# Supplementary material for: A scoping review on self-regulation and reward processing measured with gambling tasks: Evidence from the general youth population
Source: PLoS One. 2024 Apr 4;19(4):e0301539. doi: 10.1371/journal.pone.0301539 (PMC10994357; doi:10.1371/journal.pone.0301539)
Supplement: S1 File — (DOCX) [file pone.0301539.s002.docx]

# Supplementary material

**S2 Table. MEDLINE search strategy of research articles investigating the association between self-regulation and reward processing measured using a gambling task in childhood and/or adolescence in the general population.**

Database: Ovid MEDLINE(R) ALL <1946 to October 31, 2022>

Search Strategy:

--------------------------------------------------------------------------------

1 exp Executive Function/ (18854)

2 executive dysfunction.tw,ab,ti. (3213)

3 executive control*.tw,ab,ti. (4190)

4 exp Emotional Regulation/ (1952)

5 exp Self-control/ (5186)

6 self regulat*.tw,ab,ti. (14219)

7 autoregulat*.tw,ab,ti. (15530)

8 emotion* regulat*.tw,ab,ti. (11774)

9 cognit* regulat*.tw,ab,ti. (326)

10 (regulat* adj3 emotion*).tw,ab,ti. (15092)

11 (regulat* adj3 cognit*).tw,ab,ti. (3466)

12 emotion* adapta*.tw,ab,ti. (172)

13 cognit* adapta*.tw,ab,ti. (245)

14 self disciplin*.tw,ab,ti. (455)

15 self control*.tw,ab,ti. (8573)

16 emotion* control*.tw,ab,ti. (1172)

17 cognit* control*.tw,ab,ti. (8091)

18 effort* control*.tw,ab,ti. (919)

19 attent* control*.tw,ab,ti. (4718)

20 (control* adj3 emotion*).tw,ab,ti. (5220)

21 (control* adj3 cognit*).tw,ab,ti. (15665)

22 (control* adj3 effort*).tw,ab,ti. (10264)

23 (control* adj3 attent*).tw,ab,ti. (10240)

24 control capacit*.tw,ab,ti. (340)

25 control abilit*.tw,ab,ti. (931)

26 cognit* flexib*.tw,ab,ti. (3884)

27 self dysregulat*.tw,ab,ti. (11)

28 emotion* dysregulat*.tw,ab,ti. (2808)

29 cognit* dysregulat*.tw,ab,ti. (65)

30 (dysregulat* adj3 emotion*).tw,ab,ti. (3100)

31 (dysregulat* adj3 cognit*).tw,ab,ti. (355)

32 develop* regulat*.tw,ab,ti. (15378)

33 motivation* regulat*.tw,ab,ti. (184)

34 (regulat* adj3 motivation*).tw,ab,ti. (1188)

35 exp Reward/ (25818)

36 reward*.tw,ab,ti. (61504)

37 reward* process*.tw. (3012)

38 reward sensitiv*.tw. (948)

39 reward hypersensi*.tw. (21)

40 reward hyposensi*.tw. (8)

41 punishment sensitiv*.tw. (197)

42 punishment hypersensi*.tw. (1)

43 punishment hyposensi*.tw. (2)

44 exp Decision Making/ (227674)

45 decision making.ab,ti. (165896)

46 (mak* adj3 decision*).ab,ti. (213306)

47 delay discount*.ab,ti. (1785)

48 exp Risk-Taking/ (35228)

49 risk tak*.ab,ti. (7966)

50 (tak* adj3 risk*).ab,ti. (13196)

51 gambling task*.mp. (2003)

52 cambridge gambling task*.tw,ab,ti. (88)

53 CGT.tw. (945)

54 iowa gambling task*.tw,ab,ti. (1176)

55 IGT.tw. (5248)

56 balloon analogue risk task*.tw,ab,ti. (291)

57 BART.tw. (884)

58 exp Child/ (2107660)

59 (child* or stepchild* or step-child* or kid or kids or girl or girls or boy or boys or teen* or youth* or youngster* or adolescent* or adolescence or preschool* or pre-school* or kindergarten* or school* or reception or elementary or primary school* or middle school* or juvenile* or minors or p?ediatric* or PICU).ti,ab. (2283155)

60 Adolescent/ (2192327)

61 (teen* or youth* or adolescen* or juvenile* or youngster* or first-grader* or second-grader* or third-grader* or fourth-grader* or fifth-grader* or sixth-grader* or seventh-grader* or highschool* or ((secondary or high*) adj2 (school* or education))).ti,ab. (532425)

62 (meta-analysis or review literature).sh. (169858)

63 meta-analy$.tw. (235102)

64 metaanal$.tw. (2697)

65 (systematic$ adj4 (review$ or overview$)).tw. (269207)

66 meta-analysis.pt. (169858)

67 review.pt. (3018228)

68 letter.pt. (1194713)

69 historical article.pt. (368856)

70 review.ti. (608828)

71 62 or 63 or 64 or 65 or 66 or 67 or 70 (3387648)

72 68 or 69 (1555814)

73 71 not 72 (3346924)

74 exp Animals/ (25901943)

75 Humans/ (20841619)

76 74 not 75 (5060324)

77 1 or 2 or 3 or 4 or 5 or 6 or 7 or 8 or 9 or 10 or 11 or 12 or 13 or 14 or 15 or 16 or 17 or 18 or 19 or 20 or 21 or 22 or 23 or 24 or 25 or 26 or 27 or 28 or 29 or 30 or 31 or 32 or 33 or 34 (131983)

78 35 or 36 or 37 or 38 or 39 or 40 or 41 or 42 or 43 or 44 or 45 or 46 or 47 or 48 or 49 or 50 (484184)

79 51 or 52 or 53 or 54 or 55 or 56 or 57 (8365)

80 58 or 59 or 60 or 61 (4108958)

81 77 and 78 and 79 and 80 (107)

82 81 not 73 (105)

83 82 not 76 (104)

84 limit 83 to english language (104)

***************************

**S3a Table. Gambling tasks used.**

|  | *Name (N studies)* | *Type* |
| --- | --- | --- |
| **Gambling task** | Iowa Gambling Task, IGT (5) | Computerised assessment |
|  | Hungry Donkey Task, HDT (3) | Computerised assessment |
|  | Cambridge Gambling Task, CGT (2) | Computerised assessment |
|  | Children’s Gambling Task (2) | Card game |
|  | Balloon Analogue Risk Task, BART (2) | Computerised assessment |
|  | Bubblegum Analogue Risk Task for Children (2) | Computerised assessment |
|  | Preschool Gambling Task, PGT (1) | Card game |
|  | Iowa Gambling Task for Children, IGT-C (1) | Computerised assessment |

**S3b Table. Description of gambling tasks (original and adapted versions).**

| *Gambling task (N studies)* | *Original task* | *Other versions (N studies)* |
| --- | --- | --- |
| **Iowa Gambling Task, IGT (5)** | A gambling task measuring decision-making that was not originally computerised (Bechara et al., 1994), but that is now more commonly administered on a screen. The participants have to choose one card from one of the four available decks, which can be advantageous or disadvantageous. The two advantageous decks have lower rewards but with much fewer losses, thus leading to a net win, while the two disadvantageous decks have higher rewards but with much larger losses, thus leading to a net loss. Participants are told that some of decks were better than others, and to try to win as much money as possible. They are tested on their capacity to adapt and adjust their decisions over a certain number of trials (~100), and are allowed to switch between decks in order to maximise their long-term rewards. The performance on the task is assessed as the difference between the number of advantageous and disadvantageous selections across the blocks, so the dependent variable is the net score on the trials. | **Hungry Donkey Task, HDT (3):** An age-appropriate computerised version of the IGT where children are told that they have to help a hungry donkey get as many apples as possible (Crone & van der Molen, 2004). They can get the apples by using one out of four keys, each of which opens a door. When opening one door, the number of won apples is displayed in green and the number of lost apples is displayed in red. Two doors are advantageous, with one yielding frequent tiny losses and one yielding infrequent small losses; two doors are disadvantageous, with one yielding frequent small losses and one yielding infrequent large losses. Overtime, choosing the disadvantageous doors yields a net loss of 10 apples, while choosing the advantageous doors yields a net gain of 10 apples. The number of trials varies depending on the study. The performance outcome measure is the total number of trials in which a net loss occurs, or the net-score difference between advantageous and disadvantageous choices.  **Children’s Gambling Task (2):** A simplified version of the IGT to assess decision-making where children receive candies or stickers instead of a monetary reward (Kerr & Zelazo, 2004). Two decks (one advantageous and one disadvantageous) of 50 cards each are used, and either happy (win) or sad faces (loss) can be displayed. More rewards are given for the advantageous deck, but larges losses can occur across trials. With the disadvantageous deck, the opposite situation is presented. Usually, 50 trials are conducted but can also be fewer. The dependent variable is the net score on the trials, that is the number of advantageous choices minus the number of disadvantageous choices, with higher scores indicating better performance.  **Preschool Gambling Task, PGT (1):** This IGT version was created specifically for pre-schoolers and includes two variants: a trial-focus variant, where the focus is on the immediate gains and losses, and an integrated-focus variant, where the focus is on the overall outcome. The PGT comprises two decks of 50 cards. In the integrated-focus variant, the advantageous deck has an image of a chick and the disadvantageous one has an image of a giraffe, while in the trial-focus variant the advantageous deck has a cow and the disadvantageous deck has a pig. In the integrated-focus trial, the PGT is set up as a staircase in a house going from the basement (bottom) to the child’s bedroom (top), with the top floors indicating good progress, and a magnet is used to show the progress. In the trial-focus variant, a marker is used to circle the outcome of each trial. Instructions are different depending on the variant. Children were given points if they had correctly identified the deck and the reason why it was a good choice. |
| **Cambridge Gambling Task, CGT (2)** | A computerised gambling task from the Cambridge Neuropsychological Test Automated Battery (CANTAB) which assesses various aspects of decision-making (Rogers et al., 1999). The participants are explained the structure of the task prior to the start of the trial. The screen shows ten boxes, of which some are red and some are blue, and are told that a yellow token is hidden in one of these boxes. The participants have to correctly guess whether the token is hidden behind a red or a blue box. Each of the five stages entails several blocks of trials. The first stage (decision-making stage) is about making a decision about the location of the token, while the remaining stages (gambling stages) are about betting a proportion of the initially given points (~100), with the aim of winning more points. Points are gained by making correct bets, but are lost in incorrect bets. The current bet value is displayed in a circle in the centre of the screen and can incrementally increase or decrease. The six outcomes produced by the CGT are: *delay aversion*, the time participants are prepared to wait in order to place a higher or lower bet; *deliberation time*, a measure of pre-motor processing and movement time and corresponds to the mean time taken (measured in milliseconds) to make a colour box response after the decision-making information has been presented; *risk-taking*, the mean proportion of points bet on trials where the most probable colour response is made, with more risk-taking indicating a higher sensitivity to reward/lower sensitivity to punishment; *risk adjustment*, the tendency to bet more points when the likelihood of correctly guessing where the token is hidden is high; *quality of decision-making*, the mean proportion of trials where the participant bet on the most likely outcome; and, *overall proportion bet*, the mean proportion of points that are gambled across all trials. | |
| **Balloon Analogue Risk Task, BART (2)** | A computerised gambling task which measures risk-taking behaviour and one that models “real world” risk behaviour where the chances of reward or loss are balanced. In the BART, a balloon, a pump and a prize meter are displayed, and participants have to inflate the balloon to earn money (Lejuez et al., 2007). They do so by clicking a button, and the more they click the more the balloon inflates, meaning that the reward can be bigger. The participants are aware that the balloon might pop after a certain number of clicks, but they do not know the exact number. If the balloon explodes, all the money/points is/are lost. Therefore, inflating the balloon more means a higher reward, but also a higher chance of losing. Knowing this, participants can decide to stop pumping the balloon and collect the money/points, which then goes to a ‘permanent’ bank visible on the screen. The outcome measures are the total number of exploded balloons and the average number of pumps across non-exploded balloons (adjusted average of pumps) recorded during the trials (~15–30). A higher number in each of these measures corresponds to greater risk-taking. | **Bubblegum Analogue Risk Task, BART-C (2):** An adaptation of the BART made suitable for children, this version uses a similar point system but is shorter. After the child has clicked on the monkey on the screen, the monkey puffs to blow up a bubble. The more puffs, the larger the bubble and the larger the potential gain. The bubble can increase up to 50 puffs but can explode at any point. The child can also decide to stop after a certain amount of puffs and collect the points so far accumulated. Outcome measures are Total score (cumulative number of saved points), and Adjusted Puffs (average number of puffs without explosion); however, Bell et al. (2019) also introduced the Coefficient of Variability (COV; standard deviation of adjusted puffs divided by the mean of adjusted puffs), and an additional Reckless measure which is the result of risking more but earning less, and is obtained by subtracting grade-adjusted Z-score for total score from grade-adjusted Z-score for adjusted puffs. This version of the BART was adjusted so that the explosion would happen in the first trials to ensure that the child would be exposed to an early adverse outcome. |

**S4 Table. Self-regulation questionnaires/tasks used (original and other versions).**

| *Self-regulation questionnaire/task (N studies)* | *Original measure* | *Other versions (N studies)* | |
| --- | --- | --- | --- |
| *Self-report questionnaires* | | |  |
| **Child Social Behaviour Questionnaire, CSBQ (Francesconi et al., 2022; based on Hogan et al., 1992) (1)** | The CSBQ is based on the Adaptive Social Behaviour Inventory (ASBI) by Hogan et al. (1992). The scale was tested as part of a wider battery in 3 year-old children. The 30 items of the scale assessed the children’s social competence using three scales: express, comply, and disrupt. Francesconi et al. (2022) used only 10 items from the two CSBQ subscales: emotional dysregulation (5 statements) and independence self-regulation (5 statements). Children replied to the statements by indicating ‘not true’, ‘somewhat true’, and ‘certainly true’, with higher scores indicating more emotional dysregulation and more independence self-regulation, respectively. | | |
| **Emotion Dysregulation Scale for Children, EDS-C (Morrongiello et al., 2012; modelled after Gratz and Roemer, 2004; Neumann et al., 2010) (1)** | The original questionnaire is called Difficulties in Emotion Regulation Scale (DERS), developed by Gratz and Roemer (2004). The self-report questionnaire was tested in undergraduate students and consisted of 41 items asking them to say how often, on a 5-point scale, something applied to them. The aim is to assess clinically relevant difficulties in emotion regulation. It assesses several dimensions of emotion regulation, summarised in six scales: lack of emotional awareness; lack of emotional clarity; difficulties controlling impulsive behaviour when distressed; difficulties engaging in goal-directed behaviour when distressed; nonacceptance of negative emotional responses; and limited access to emotion regulation strategies. Neumann et al. (2010) used a very similar version (36 items instead of 41) to validate it in adolescents aged 11-17 years. The EDS-C by Morrongiello et al. (2012) comprised three subscales of a total of 19 items that could be completed by 8–10-year-old children: difficulties engaging in goal-directed behaviour, impulse control difficulties, and limited access to emotion regulation strategies. The answers were rated on a 5-point scale where higher scores meant greater emotional dysregulation. | | |
| *Cognitive assessments* | | |  |
| **NIH Toolbox Dimensional Change Card Sort, DCCS (Byrne et al., 2021; Harms et al., 2014; Hongwanishkul et al., 2016; originally developed by Frye et al., 1995, and Zelazo, 2006) (3)** | The DCCS is a measure of executive function developed by Frye et al. (1995). It can be used at all ages, however, the version described in Zelazo (2006) mainly focused on preschool children. The standard version comprises two target cards (a blue rabbit and a red boat) which are shown to the children, who have to sort bivalent cards (e.g., red rabbit and blue boat) depending on one dimension (e.g., colour). During a post-switch phase, they are told to sort the same types of cards according to the other dimension (e.g., shape). The DCCS can be used to identify differences in the development of executive function in the preschool years. This measure assesses **flexible rule use** and accuracy, and reaction time (in more complex versions of the tool). In the standard version, the game is successfully completed if the children correctly sort at least 9 cards out of 12. In Byrne et al. (2021), the DCCS was used to assess **cognitive flexibility (or set-shifting)** and was administered as a computerised task of 4 minutes. In Harms et al. (2014), the task was also computerised and participants could practise beforehand by completing 40 practice trials, followed by the real 40-trial task where 75% of the trials were focused on the shape and 25% of the trials were about the colour. The task measured the **shifting/updating** aspects of executive function. Finally, in Hongwanishkul et al. (2016) the DCCS is also referred to as a measure of **flexible rule use**, and both the standard and a more difficult (“border”) versions were used. A score ranging from 0 to 3 was given based on the level of performance (3 was given if children successfully passed both versions of the DCCS). | | |
| **Stroop Color and Word Test/Task (Lamm et al., 2006; Poon, 2018; Prencipe et al., 2011; originally developed by Stroop, 1935) (3)** | The Stroop Task is a neuropsychological test originally developed by Stroop in 1935 and is considered to be a measure of **selective attention** and **response inhibition**. It has been widely used to assess the **ability to inhibit cognitive inference**, i.e. the processing of a specific stimulus feature does not allow for the processing of a second stimulus attribute at the same time, and this is known as the Stroop Effect. Participants are presented with a series of words with three colours (red, blue, and green) using two laminated cards of 21 words each, and need to name the colour of each word that they see. There are five conditions: congruent colour word, incongruent colour word (e.g. colour red for word ‘blue’), negative word, neutral word (e.g. neutral, non-colour word such as ‘book’) and positive word. Participants need to name the colour as fast and as accurately as possible. Adjusted reaction time scores are created by subtracting twice the average time per word for each error from the total reaction time per condition. The outcome measure can be either the response latency (i.e. time in milliseconds between stimulus onset and response), or an interference score (i.e. difference in response latencies between incongruent and congruent stimuli). In Lamm et al. (2006) and Poon (2018) only the interference score was used and it was meant to measure **inhibitory ability**. In Prencipe et al. (2011) only the congruent and incongruent conditions were used, and lower scores indicated better performance. | **Fruit Stroop Task (Groppe and Elsner, 2017, 2015; originally developed by Archibald and Kerns, 1999, and used by Roebers et al., 2011) (2):** This adapted version of the Stroop task was developed by Archibald and Kerns (1999) and has also been used by Roebers et al. (2011). Both studies were cited by Groppe and Elsner (2017, 2015), and the task was used to assess **inhibition**. Children were presented with 4 pages with 25 stimuli each: coloured rectangles, four kinds of fruits/vegetables with appropriate colours, the same fruit/vegetables in grey, and the same fruit/vegetables with the wrong colour. Children had to name, as fast as possible, the colour of each item or fruit/vegetable. The time in seconds was recorded and a measure of interference was created, with higher values indicating more interference (i.e. lower ability to inhibit the dominant response of naming the colour that the item is supposed to have).  **Counting Stroop (Romer et al., 2009; previously used by Bush et al., 1998) (1):** Another adapted version of the Stroop task, previously used by Bush et al. (1998). Romer et al. (2009) administered a computerised version of this task to assess **cognitive control**. Participants were required to sort cards according to one or two conditions (congruent and incongruent). The screen displays one card at the time showing 1 to 5 instances of a digit. In the congruent condition, they need to sort the cards according to the digit as quickly as possible (e.g. three “2’s” go into the “2” pile). In the incongruent condition, they need to sort the cards depending on the number of digits displayed on the card (e.g. three “2’s” go into the “3” pile). The dependent variable is the reaction time difference score between the two conditions. | |
| **Flanker Test of Focused Attention (Imal et al., 2020; Romer et al., 2009; originally developed by Eriksen and Eriksen, 1974) (2)** | The Flanker task was originally developed by Eriksen and Eriksen (1974) and is considered to be a measure of **selective attention**, **information processing**, and **inhibitory function**. It requires participants to press a left-hand or right-hand response key according to the direction of the arrow displayed at the centre of the screen. Four arrows flank the central arrow, and can display the same direction (congruent condition) or the opposite direction (incongruent condition). The incongruent trials require cognitive control. Participants have to mentally ignore the flanking arrows. The main outcome measure is the difference in reaction time between congruent and incongruent trials. Romer et al. (2009) followed the original procedure, while Imal et al. (2020) followed the one outlined by the National Institute of Health Toolbox, with 29 congruent trials and 17 incongruent trials. Trials were valid depending on a number of factors, including whether the reaction times were too slow or too fast. The main performance measures were the percentage of correct responses in incongruent trials and the mean reaction time of correct incongruent trials. | **Attention network task (Harms et al., 2014; developed by Rueda et al., 2004) (1):** The Attention Network Test developed by Rueda et al. (2004) is a child-friendly version of the Flanker task created to study the development of **attentional networks** in children. Instead of arrows, in this task fish are displayed using the same structure as in the Flanker task. Congruent and incongruent conditions are equally used, but a spatial cue is also displayed 150ms before the central fish appears, and a practice block of 24 trials was done (and feedback was provided) before the real test, which consisted of four blocks of 48 trials. The scores were the mean accuracy and the median reaction times for both congruent and incongruent trials. | |
| **Trail Making Test, TMT-A & TMT-B (Gonzalez-Gadea et al., 2015; Smith et al., 2012; originally used by Reitan, 1955, and by Spreen and Gaddes, 1969) (2)** | The Trail Making Test, which was originally part of the Army Individual Test of General Mental Ability in 1944 (Spreen and Gaddes, 1969), was first used by Reitan in 1955 for neuropsychological testing. Reitan (and subsequently Spreen and Gaddes) used the intermediate version, whereby the participant is required to first link numbers on a page in the correct numerical order (TMT-A), and to then do the same but also with letters (numbers and letters have to be linked and alternated by drawing connecting lines; TMT-B). Participants need to do this as quickly as possible. The score is the time in seconds taken by the participants for each of the two parts. Gonzalez-Gadea et al. (2015) included both parts: TMT-A was used to assess **attention** and **processing speed**, while TMT-B was used to assess **set-shifting (or cognitive flexibility)**. Instead, Smith et al. (2012) used only TMT-B and described it as a task requiring **set-shifting**, **working memory**, and **inhibition** of a previously correct response. | | |
| **Cognitive Flexibility Task (Groppe & Elsner, 2017; Groppe & Elsner, 2018; Roebers et al., 2011; Zimmermann et al., 2022) (2)** | The Cognitive Flexibility Task was originally developed by Zimmermann et al. (2002) and then adapted by Roebers et al. (2011). Two different fish (one single-coloured and one multi-coloured) are displayed on a screen at the same time, one on the left and one on the right. In each trial, the side where the fish are randomly changes. Participants were required to feed both fish consecutively by pressing the two corresponding keys. This task requires that the participant remembers which type of fish they had fed in the previous trial. The outcome measure is the proportion of correct responses in the trials. Roebers et al. (2011) used 46 trials. In Groppe and Elsner (2017, 2015), who used the task to assess **attention shifting**, the outcome measure was the number of correct responses in 22 switch-trials (when the child had to change their response pattern from right-left to right-right or left-left reactions). | | |
| **Wisconsin Card Sort Test, WCST (Smith et al., 2012; originally developed and used by Berg, 1948; Grant and Berg, 1948; Heaton et al., 1993) (1)** | The WCST was originally developed and used by Grant and Berg (1948), and the manual was then revised and expanded by Heaton et al. (1993). The original test assessed **set-shifting** and **abstract behaviour** (i.e. the ability of being flexible despite changing reinforcement), and included 60 response cards, each containing one to four identical figures (either stars, crosses, triangles, or circles) of a single colour (either red, yellow, blue, or green). The cards could be sorted according to the number, the shape, or the colour of the figures. There were also four stimulus cards: one red triangle, two green stars, three yellow crosses, and four blue circles. These stimulus cards were displayed in front of the participant in this order from left to right, and the participant had to sort the response cards depending on colour, number, or shape, without knowing which one of these was correct (the experimenter would tell the participant when they were right or wrong). After five correct consecutive responses, the sorting category was changed to one of the unused categories. This was done until all the categories were used, and then the cycle was repeated. The experiment was concluded once the participant had completed nine categories correctly. Smith et al. (2012) used a computerised version of this test, where a stimulus card is displayed on a screen and the participant has to match the card to one of four key cards. No explanation as to how to sort the cards is given to the participant. As the sorting rules change during the test, the participant has to adapt depending on the feedback received. The score was the number of mistakes made, and the outcome measure was perseverative error scores (considered as standard for the evaluation of WCST performance). | | |
| **Contingency Naming Test, CNT (Poon, 2018; Taylor et al., 1987) (1)** | The CNT was originally developed by Taylor et al. (1987) as a measure of **mental set switching** and **rapid memory retrieval abilities**, and was modelled after the Stroop Color and Word Test (Stroop, 1935). Poon (2018) states that there is general consensus that the CNT assesses **attentional control** and **cognitive flexibility**. The participants are presented with a stimulus set of nine practice items and 27 test items. The stimulus has a different shape (circle, triangle, or square), a different colour (blue, yellow, or red), and a smaller independent shape (also either a circle, triangle, or square) embedded inside the big shape. There can be a backwards arrow displayed above the big shape for some of the stimuli. In level A (**attentional control**), participants need to name the colour or shape as quickly as possible, while in level B (**cognitive flexibility**), participants need to switch between naming the colour and shape depending on one or two attributes. The CNT does not require the participant to suppress automatic responses, as these are equally salient responses and the participant can choose which one is correct. | | |
| **Shifting task (Garon et al., 2014; Garon N. et al., 2022; from the tasks developed by Hughes, 1998, and Zelazo, 2006) (1)** | The shifting task was developed by Garon et al. (2014) by combining aspects of the Teddy Bear task (Hughes, 1998) and the DCCS (Zelazo, 2006). This task was used to measure **shifting**. In the preshift phase, children need to find the animal hidden under one or two flaps different in shape. When using two dimensions, colour is also added to the shapes, however, children have to ignore the colour and only focus on the shape (selective attention). In the postshift phase, positive and negative priming trials are used: in negative priming trials, children have to stop ignoring the colour (meaning that they start matching based on the colour); in positive priming trials, children have to match based on the colour too, but this time the colour is different. The child received a score of 1 when they lifted the correct flap, and a score of 0 for the incorrect flap. In Garon et al. (2022), the preshift phase had 10 trials, and if children scored at least 6 out 10 they passed to the postshift phase (10 trials), with scores ranging from 0 to 20. | | |
| **Conners’ continuous performance test, CPT-II (Smith et al., 2012; developed by Conners, 2002, based on Rosvold et al., 1956) (1)** | The CPT-II is based on the CPT originally described by Rosvold et al. (1956). The CPT-II is administered on a computer and is a go/no-go task used to assess **motor inhibition/impulsivity** as well as **sustained attention**. A random letter is flashed on the screen at varying intervals between 1 and 4 seconds long. Participants need to press a key as fast as possible when they see the letter, however, they need to refrain from pressing the key when they see the non-target letter ‘X’. Outcome measures can include omissions, commission and perseveration errors, hit reaction time, hit reaction time standard error, variability, and detectability [38]. Smith et al. (2012) used CPT-II as a measure of **speed of processing** and **attention**, and commission and perseveration errors were the outcome measures. | | |
| **Battersea Multitask Paradigm, BMP (Gonzalez-Gadea et al., 2015; developed by Mackinlay et al., 2006; originally designed by Burgess et al., 2000) (1)** | A multitask test that was developed by Mackinlay et al. (2006) specifically for children (the original adult version was designed by Burgess et al., 2000). The BMP comprises three interleaved tasks to be completed within three minutes: fruit sorting into boxes of different sizes, caterpillar (different sizes) colouring on a paper using crayons, and counter sorting from a large tub onto grids of different sizes. Children need to follow a set of rules, with the goal of gaining points without breaking the rules. In the study conducted by Gonzalez-Gaeda et al. (2015), the BMP assesses **set-shifting** (number of tasks attempted), **strategy formation** (strategy performance), and **inhibitory control** (rule-breaking behaviour or number of errors); however, because children were assessed on their ability to generate a plan before starting, **planning** **abilities** were also measured. Given the scope of this review, only **planning abilities** and task attempted (i.e. **cognitive flexibility**) were included. | | |
| **Tower of London (Poland et al., 2016; based on Shallice, 1982) (1)** | A planning task developed by Shallice (1982) used to assess **planning skills**. Participants are presented with 12 test problems, which need to be resolved in a specified number of moves while moving one bead at the time. In the version used by Poland et al. (2016), children were allowed two minutes to solve each problem. 2 points were given if the problem was solved during the first attempt, 1 point for the second attempt, and 0 points if they failed, with the final scores ranging between 0 and 24 points. | **Stockings of Cambridge [12] (1):** A task from the CANTAB originally used by Hughes et al. (1994) which measures **goal-setting** and **(spatial) planning ability**. Three stockings are displayed, each of them containing one ball. Different patterns are created in two different displays. Participants need to use problem-solving strategies to match the pattern shown in one display by moving the balls on the other display by making as few moves as possible. Outcome measures: difficulty level; mean moves used; thinking time. | |
| **Hearts and Flowers Task (Ursache and Raver, 2015; from Davidson et al., 2006; Diamond et al., 2007) (1)** | Originally developed by Davidson et al. (2006) and previously called the Dots Task (Diamond et al., 2007), this task measures three executive functions simultaneously: **attention set-shifting**, **inhibition**, and **working memory**. Two stimuli (hearts and flowers) are displayed, and participants need to press the button on the side of the heart when this appears, and the button on the side of the flower when they see a flower. In Ursache and Raver (2015), participants were first presented with only hearts (12 trials), then with only flowers (12 trials), and finally with mixed hearts and flowers (33 trials). The difference in response latency was calculated by subtracting the mean latency on the trials with hearts only from the mean latency on the mixed hearts/flowers trials, and a lower difference meant higher overall executive functioning. | | |

# References

1. Francesconi M, Flouri E, Harrison A. Decision-making difficulties mediate the association between poor emotion regulation and eating disorder symptoms in adolescence. Psychol Med. 2022; 1–10. doi:10.1017/S003329172200037X

2. Hogan AE, Scott KG, Bauer CR. The Adaptive Social Behavior Inventory (ASBI): A new assessment of social competence in high-risk three-year-olds. J Psychoeduc Assess. 1992;10: 230–239.

3. Morrongiello BA, Kane A, McArthur BA, Bell M. Physical risk taking in elementary-school children: Measurement and emotion regulation issues. Personal Individ Differ. 2012;52: 492–496. doi:10.1016/j.paid.2011.11.003

4. Gratz KL, Roemer L. Multidimensional assessment of emotion regulation and dysregulation: Development, factor structure, and initial validation of the difficulties in emotion regulation scale. J Psychopathol Behav Assess. 2004;26: 41–54.

5. Neumann A, van Lier PA, Gratz KL, Koot HM. Multidimensional assessment of emotion regulation difficulties in adolescents using the difficulties in emotion regulation scale. Assessment. 2010;17: 138–149.

6. Byrne ME, Tanofsky-Kraff M, Lavender JM, Parker MN, Shank LM, Swanson TN, et al. Bridging executive function and disinhibited eating among youth: A network analysis. Int J Eat Disord. 2021;54: 721–732. doi:10.1002/eat.23476

7. Harms MB, Zayas V, Meltzoff AN, Carlson SM. Stability of executive function and predictions to adaptive behavior from middle childhood to pre-adolescence. Front Psychol. 2014;5. doi:10.3389/fpsyg.2014.00331

8. Hongwanishkul D, Happaney KR, Lee WS, Zelazo PD. Assessment of hot and cool executive function in young children: Age-related changes and individual differences. Measurement of Executive Function in Early Childhood. Psychology Press; 2016. pp. 617–644.

9. Frye D, Zelazo PD, Palfai T. Theory of mind and rule-based reasoning. Cogn Dev. 1995;10: 483–527.

10. Zelazo PD. The Dimensional Change Card Sort (DCCS): A method of assessing executive function in children. Nat Protoc. 2006;1: 297–301.

11. Lamm C, Zelazo PD, Lewis MD. Neural correlates of cognitive control in childhood and adolescence: disentangling the contributions of age and executive function. Neuropsychologia. 2006;44: 2139–48.

12. Poon K. Hot and cool executive functions in adolescence: Development and contributions to important developmental outcomes. Front Psychol. 2018;8. doi:10.3389/fpsyg.2017.02311

13. Prencipe A, Kesek A, Cohen J, Lamm C, Lewis MD, Zelazo PD. Development of hot and cool executive function during the transition to adolescence. J Exp Child Psychol. 2011;108: 621–37. doi:10.1016/j.jecp.2010.09.008

14. Stroop JR. Studies of interference in serial verbal reactions. J Exp Psychol. 1935;18: 643.

15. Groppe K, Elsner B. Executive function and weight status in children: A one-year longitudinal perspective. Child Neuropsychol. 2017;23: 129–147.

16. Groppe K, Elsner B. The influence of hot and cool executive function on the development of eating styles related to overweight in children. Appetite. 2015;87: 127–136. doi:10.1016/j.appet.2014.12.203

17. Archibald SJ, Kerns KA. Identification and description of new tests of executive functioning in children. Child Neuropsychol. 1999;5: 115–129.

18. Roebers CM, Röthlisberger M, Cimeli P, Michel E, Neuenschwander R. School enrolment and executive functioning: A longitudinal perspective on developmental changes, the influence of learning context, and the prediction of pre-academic skills. Eur J Dev Psychol. 2011;8: 526–540.

19. Romer D, Betancourt L, Giannetta JM, Brodsky NL, Farah M, Hurt H. Executive cognitive functions and impulsivity as correlates of risk taking and problem behavior in preadolescents. Neuropsychologia. 2009;47: 2916–2926.

20. Bush G, Whalen PJ, Rosen BR, Jenike MA, McInerney SC, Rauch SL. The counting Stroop: an interference task specialized for functional neuroimaging—validation study with functional MRI. Hum Brain Mapp. 1998;6: 270–282.

21. Imal A.E., O’leary S., Wexler B.E. Risk-taking patterns of children, associated cognitive weaknesses, and prevention of negative outcomes. Psychiatr Res Clin Pract. 2020;2: 34–40. doi:10.1176/appi.prcp.2020.20190020

22. Eriksen BA, Eriksen CW. Effects of noise letters upon the identification of a target letter in a nonsearch task. Percept Psychophys. 1974;16: 143–149.

23. Rueda MR, Fan J, McCandliss BD, Halparin JD, Gruber DB, Lercari LP, et al. Development of attentional networks in childhood. Neuropsychologia. 2004;42: 1029–1040.

24. Gonzalez-Gadea ML, Scheres A, Tobon CA, Damm J, Baez S, Huepe D, et al. Stop Saying That It Is Wrong! Psychophysiological, Cognitive, and Metacognitive Markers of Children’s Sensitivity to Punishment. PloS One. 2015;10: e0133683. doi:10.1371/journal.pone.0133683

25. Smith DG, Xiao L, Bechara A. Decision making in children and adolescents: impaired Iowa Gambling Task performance in early adolescence. Dev Psychol. 2012;48: 1180–7. doi:10.1037/a0026342

26. Reitan RM. The relation of the trail making test to organic brain damage. J Consult Psychol. 1955;19: 393.

27. Spreen O, Gaddes W. Development norms for 15 neuropsychological tests age 6 to 15. Cortex J Devoted Study Nerv Syst Behav. 1969.

28. Zimmermann P, Gondan M, Fimm B. KiTAP-A Test Battery of Attentional Performance for Children. Vera Fimm Psychol Testsysteme Herzogenrath Ger. 2002.

29. Berg EA. A simple objective technique for measuring flexibility in thinking. J Gen Psychol. 1948;39: 15–22.

30. Grant DA, Berg E. A behavioral analysis of degree of reinforcement and ease of shifting to new responses in a Weigl-type card-sorting problem. J Exp Psychol. 1948;38: 404.

31. Heaton R, Chelune G, Talley J, Kay G, Curtiss G. Wisconsin card sorting test manual: revised and expanded. Psychol Assess Resour. 1993; 5–57.

32. Taylor HG, Albo VC, Phebus CK, Sachs BR, Bierl PG. Postirradiation treatment outcomes for children with acute lymphocytic leukemia: Clarification of risks. J Pediatr Psychol. 1987;12: 395–411.

33. Garon N, Smith IM, Bryson SE. A novel executive function battery for preschoolers: Sensitivity to age differences. Child Neuropsychol. 2014;20: 713–736.

34. Garon N., Hecker O., Kwan A., Crocker T.A., English S.D. Integrated versus trial specific focus improves decision-making in older preschoolers. Child Neuropsychol. 2022. doi:10.1080/09297049.2022.2063269

35. Hughes C. Executive function in preschoolers: Links with theory of mind and verbal ability. Br J Dev Psychol. 1998;16: 233–253.

36. Conners K. Technical guide and software manual. North Tonawanda NY Multi Health Syst Conners’ Contin Perform Test CPT-II. 2002; 43–52.

37. Rosvold HE, Mirsky AF, Sarason I, Bransome Jr ED, Beck LH. A continuous performance test of brain damage. J Consult Psychol. 1956;20: 343.

38. Zane KL, Gfeller JD, Roskos PT, Bucholz RD. The clinical utility of the Conners’ Continuous Performance Test-II in traumatic brain injury. Arch Clin Neuropsychol. 2016;31: 996–1005.

39. Mackinlay R, Charman T, Karmiloff-Smith A. High functioning children with autism spectrum disorder: A novel test of multitasking. Brain Cogn. 2006;61: 14–24.

40. Burgess PW, Veitch E, de Lacy Costello A, Shallice T. The cognitive and neuroanatomical correlates of multitasking. Neuropsychologia. 2000;38: 848–863.

41. Poland SE, Monks CP, Tsermentseli S. Cool and hot executive function as predictors of aggression in early childhood: Differentiating between the function and form of aggression. Br J Dev Psychol. 2016;34: 181–197. doi:10.1111/bjdp.12122

42. Shallice T. Specific impairments of planning. Philos Trans R Soc Lond B Biol Sci. 1982;298: 199–209.

43. Hughes C, Russell J, Robbins TW. Evidence for executive dysfunction in autism. Neuropsychologia. 1994;32: 477–492.

44. Ursache A, Raver CC. Iowa Gambling Task performance and executive function predict low-income urban preadolescents’ risky behaviors. Personal Individ Differ. 2015;79: 1–6. doi:10.1016/j.paid.2015.01.010

45. Davidson MC, Amso D, Anderson LC, Diamond A. Development of cognitive control and executive functions from 4 to 13 years: Evidence from manipulations of memory, inhibition, and task switching. Neuropsychologia. 2006;44: 2037–2078.

46. Diamond A, Barnett WS, Thomas J, Munro S. Preschool program improves cognitive control. Science. 2007;318: 1387–1388.
